# Relapsing Eczema

**Published:** 1908-09-12

**Authors:** 


### DERMATOLOGY.

### RELAPSING ECZEMA.

A boy, aged ten, was said by his mother to have had a " breaking-out " in front of his elbow off and on since he was three months old. It would sometimes fade away altogether, but this time it had lasted for over a month, and it kept on " discharging matter." There were eight other children in the family, but none of them had had anything wrong with their skins. On examination there was an irregularly shaped patch of dermatitis, the size of a crown-piece, occupying the centre of the right antecubital space. Its margins were ill-defined, and the surface was marked by pustules here and there, and also by a deep fissure running parallel to the fold of the elbow. No " apple-jelly nodules " could be seen on careful pressure with the diascope. The front of the left elbow was affected very slightly in a similar manner, and there were a few patches of dry, squamous eczema around the mouth. There was no general xerodermia, and the boy's general health was unimpaired. At first sight the main patch of disease somewhat resembled a lupus exedens —hence the use of a watch-glass to press out the blood from its margins with a view to the discovery of any tuberculous foci. If undue attention had been paid to the history the duration of the disease might have influenced us in favour of lupus vulgaris. This latter affection is more likely to be mistaken for an eczema than *vice versa*, but the former is the more serious error. One sometimes sees cases of lupus upon the nose which, in quite the early stage, resemble an impetiginous eczema very closely. In such a case the use of the diascope is essential, and it will be pretty sure to reveal the presence of the yellowish-brown points or nodules that are so characteristic of the disease. Moreover, the colour of lupus is of a darker red than eczema, and, by the sense of touch, it is felt to be more infiltrated. In the present case it was only after severe cross-examination that the fact of the complete disappearance of the lesion at certain times was elicited, and this phenomenon alone would, of course, have sufficed to distinguish it from lupus. Then we had the presence of the dry patches around the mouth and the condition of the other elbow to guide us. Here, then, was a case of flexural eczema of a relapsing character in a boy apparently in the best of health, but inquiry into the state of his gastro-intestinal apparatus showed that the starchy element was largely in excess in his diet, and that there was a tendency to chronic constipation. In many forms of chronic or relapsing eczema it is quite common for patients to volunteer the remark that they always get an outbreak or an exacerbation when the bowels are costive. This boy was given a weak cyllin lotion for bathing the affected part, and an ointment consisting of five minims of ichthyol in Lassar's paste, half strength. When the inflammatory condition had subsided a little salicylic acid would then be added to the ointment.

